# Mitotic gene regulation by the N-MYC-WDR5-PDPK1 nexus

**DOI:** 10.1186/s12864-024-10282-6

**Published:** 2024-04-11

**Authors:** Sarah A. Streeter, Alexandria G. Williams, James R. Evans, Jing Wang, Alissa D. Guarnaccia, Andrea C. Florian, Rafet Al-Tobasei, Qi Liu, William P. Tansey, April M. Weissmiller

**Affiliations:** 1 Department of Biology, Middle Tennessee State University, Murfreesboro, TN 37132 USA; 2 Center for Quantitative Sciences, Vanderbilt University Medical Center, Nashville, TN 37232 USA; 3 Department of Biostatistics, Vanderbilt University Medical Center, Nashville, TN 37203 USA; 4 Department of Cell and Developmental Biology, Vanderbilt University School of Medicine, Nashville, TN 37240 USA; 5 Department of Computer Science, Middle Tennessee State University, Murfreesboro, TN 32132 USA; 6 Department of Biochemistry, Vanderbilt University School of Medicine, Nashville, TN 37240 USA; 7 Present Address: Department of Discovery Oncology, Genentech Inc, South San Francisco, CA 94080 USA; 8 Present Address: Department of Biology, Belmont University, Nashville, TN 37212 USA

**Keywords:** N-MYC, WDR5, PDPK1, Mitosis, Chromosome segregation, Spindle pole, Genomic instability, Neuroblastoma

## Abstract

**Background:**

During mitosis the cell depends on proper attachment and segregation of replicated chromosomes to generate two identical progeny. In cancers defined by overexpression or dysregulation of the *MYC* oncogene this process becomes impaired, leading to genomic instability and tumor evolution. Recently it was discovered that the chromatin regulator WDR5—a critical MYC cofactor—regulates expression of genes needed in mitosis through a direct interaction with the master kinase PDPK1. However, whether PDPK1 and WDR5 contribute to similar mitotic gene regulation in MYC-overexpressing cancers remains unclear. Therefore, to characterize the influence of WDR5 and PDPK1 on mitotic gene expression in cells with high MYC levels, we performed a comparative transcriptomic analysis in neuroblastoma cell lines defined by *MYCN*-amplification, which results in high cellular levels of the N-MYC protein.

**Results:**

Using RNA-seq analysis, we identify the genes regulated by N-MYC and PDPK1 in multiple engineered CHP-134 neuroblastoma cell lines and compare them to previously published gene expression data collected in CHP-134 cells following inhibition of WDR5. We find that as expected N-MYC regulates a multitude of genes, including those related to mitosis, but that PDPK1 regulates specific sets of genes involved in development, signaling, and mitosis. Analysis of N-MYC- and PDPK1-regulated genes reveals a small group of commonly controlled genes associated with spindle pole formation and chromosome segregation, which overlap with genes that are also regulated by WDR5. We also find that N-MYC physically interacts with PDPK1 through the WDR5-PDPK1 interaction suggesting regulation of mitotic gene expression may be achieved through a N-MYC-WDR5-PDPK1 nexus.

**Conclusions:**

Overall, we identify a small group of genes highly enriched within functional gene categories related to mitotic processes that are commonly regulated by N-MYC, WDR5, and PDPK1 and suggest that a tripartite interaction between the three regulators may be responsible for setting the level of mitotic gene regulation in N-MYC amplified cell lines. This study provides a foundation for future studies to determine the exact mechanism by which N-MYC, WDR5, and PDPK1 converge on cell cycle related processes.

**Supplementary Information:**

The online version contains supplementary material available at 10.1186/s12864-024-10282-6.

## Background

During mitosis a cell undergoes an ordered series of phases that allow it to divide into two daughter cells containing identical genetic material. This is a highly regulated process that relies on proper attachment of replicated chromosomes to the microtubules that make up the spindle pole prior to chromosome segregation. Any errors in chromosome attachment are detected by the spindle assembly checkpoint (SAC), which when activated can pause cells in mitosis until all chromosomes are attached correctly [1]. Proper chromosome segregation is achieved by a wide array of proteins, including kinetochore protein complexes that are responsible for attaching and aligning chromosomes to the spindle pole and other SAC signaling proteins that are recruited to sites of kinetochore formation [1, 2]. The delay on cell cycle progression induced by SAC can be long in duration or transient, with some cells being able to exit mitosis through mitotic slippage or adaptation [3]. Completion of mitosis in the absence of the SAC does not guarantee cell progeny will contain chromosomal abnormalities, but it does dramatically increase the likelihood of genomic instability and acquisition of the hallmarks of cancer [3, 4].

Genomic instability feeds into all stages of malignancy including tumorigenesis, metastasis, and tumor evolution [5]. Maintenance of genomic instability in cancers is accomplished in part through the loss or gain of chromosomes during improper mitosis, resulting in chromosomal instability and aneuploidy [6]. Cancers that show overexpression or dysregulation of the *MYC* oncogene are particularly prone to genomic instability due to the prominent effect that MYC has as an oncoprotein transcription factor in facilitating expression of genes needed for cell cycle phase progression and mitotic cell fate [7]. In non-transformed epithelial cells, overexpression of MYC delays exit from mitosis through induction of the SAC [8]. However, to overcome any chromosomal segregation delays, MYC binds to and increases expression of genes needed in spindle and kinetochore formation, allowing for premature exit from mitosis that ultimately leads to chromosomal instability [8]. These data provide an interesting molecular explanation for how cells with high levels of MYC can effectively override paused mitosis to continue cell division at the expense of increased genomic instability [9].

Beyond MYC, signaling proteins such as the Aurora Kinases and Polo-like kinases can regulate expression of genes required for chromosomal segregation and spindle pole/kinetochore formation [10], suggesting an interplay between various transcriptional regulators to influence the overall ability of cancer cells to complete mitosis. In recent years, the chromatin-regulatory protein known as WDR5 (WD repeat-containing protein 5) has also entered into the realm of proteins that can regulate cell cycle progression, with WDR5 possessing a non-chromatin role during mitosis in recruitment of specific kinesins to the spindle pole [11] and a chromatin-based role during interphase in regulation of genes needed for mitosis, including spindle and kinetochore genes [12]. The latter chromatin-based role is thought to involve a direct interaction between WDR5 and the master kinase PDPK1 (3-phosphoinositide-dependent protein kinase 1, also called PDK1). Inhibition of the WDR5-PDPK1 interaction either by chemical or genetic methods directly changes transcription of spindle and kinetochore genes, suggesting that the WDR5-PDPK1 interaction functions to regulates expression of mitotic genes [12]. However, these studies were founded in cells lacking high levels of MYC and as WDR5 is a critical cofactor for c- and N-MYC [13, 14] target gene recognition, it is worth understanding the extent by which MYC, PDPK1, and WDR5 influence expression of mitotic genes in a more relevant MYC-driven cancer context.

Here, we create a variety of genetically engineered N-MYC amplified CHP-134 cell lines to probe the gene networks regulated by N-MYC, PDPK1, and WDR5. Using RNA-seq analysis, we identify the genes regulated by N-MYC and PDPK1 and compare them to previously published gene expression data collected in CHP-134 cells following inhibition of WDR5. We find that while N-MYC regulates thousands of diverse genes in the CHP-134 genome, PDPK1 regulates specific sets of genes involved in development, signaling, and mitosis. Comparison between N-MYC- and PDPK1-regulated genes reveals that the genes commonly regulated are enriched within biological functions connected to spindle pole formation and the mitotic phase of cell cycle and overlap with genes that are also controlled by WDR5. Interestingly, in multiple N-MYC amplified cell lines, N-MYC can physically interact with PDPK1 at least in part through the WDR5-PDPK1 interaction, suggesting that a tripartite complex between the three regulators may be responsible for setting the level of mitotic gene regulation in N-MYC amplified cell lines. Overall, this study highlights the intricate and complex nature of mitotic gene regulation in cancer cells and provides a foundation for further studies in MYC-amplified cancers.

## Results

### N-MYC regulates genes involved in cell cycle in CHP-134 cells

**Fig. 1:**
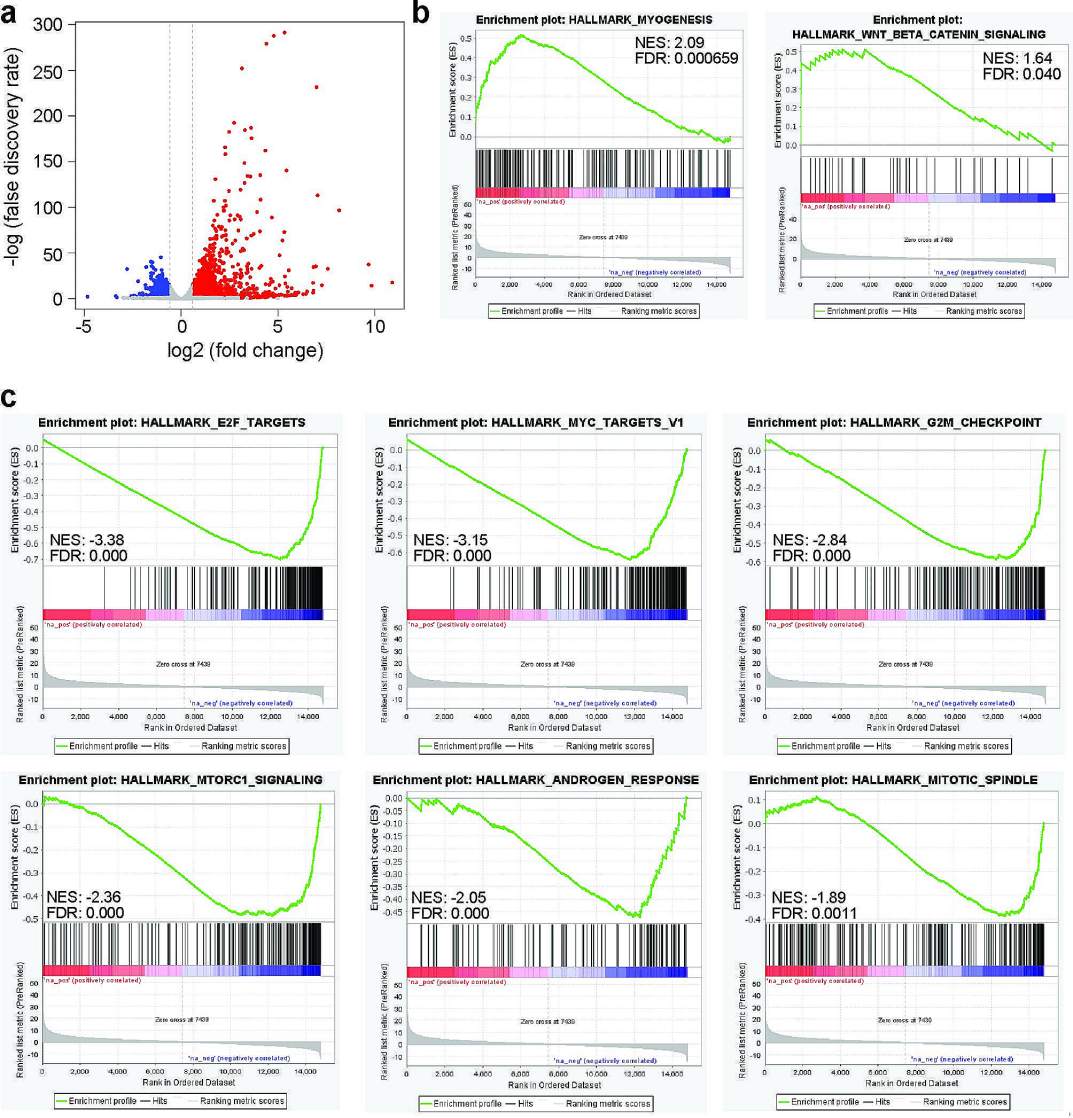
Induction of OmoMYC causes widespread gene expression changes. (**a**) Volcano plot showing data for all genes analyzed following induction of OmoMYC versus EGFP in engineered CHP-134 cells for three days. Blue dots indicate genes decreased in expression by a fold change of < -1.5 and FDR < 0.05. Red dots indicate genes that increased in expression by a fold change of > 1.5 and FDR < 0.05. (**b**) Gene set enrichment analysis was performed to compare OmoMYC-induced changes in gene expression against the MSigDB Hallmark data sets. Genes that increased in expression are significantly enriched among the gene categories shown. (**c**) Gene set enrichment analysis was performed to compare OmoMYC-induced changes in gene expression against the MSigDB Hallmark data sets. Genes that decreased in expression are significantly enriched among the gene categories shown. Additional gene categories are noted in Additional File 2: Table S2

MYC has been shown to regulate mitotic gene expression in a variety of cell lines [15]. To investigate whether this is true in N-MYC amplified cells, we engineered the N-MYC amplified CHP-134 cell line so that we could induce expression of either enhanced green fluorescent protein (EGFP) or the genetic MYC inhibitor known as OmoMYC through the Tet-On system that we previously characterized [16]. Induction of OmoMYC compared to EGFP followed by RNA-seq analysis reveals that inhibiting N-MYC in this manner causes thousands of gene expression changes in either direction (Fig. 1a, Additional File 1: Table S1). To categorize genes regulated by N-MYC, we performed gene set enrichment analysis (GSEA) against the MSigDB Hallmark data sets (Additional File 2: Table S2). Genes that are induced by OmoMYC induction were enriched significantly among only two data sets, including myogenesis and WNT-signaling pathway (false-discovery rate (FDR) < 0.05) (Fig. 1b, Additional File 2: Table S2) suggesting that induced genes do not cluster within any particular biological process. In contrast, genes that are suppressed by OmoMYC induction were enriched among a large group of data sets, with those related to cell cycle function (“E2F targets”, “G2/M Checkpoint”, and “Mitotic Spindle”) being among the top data sets based on FDR (Fig. 1c, Additional File 2: Table S2). These data indicate that consistent with known functions of MYC proteins, N-MYC promotes expression of a vast number of genes in N-MYC amplified cells that impact important cellular processes related to growth and cell division.

### PDPK1 facilitates expression of mitotic genes in CHP-134 cells

**Fig. 2:**
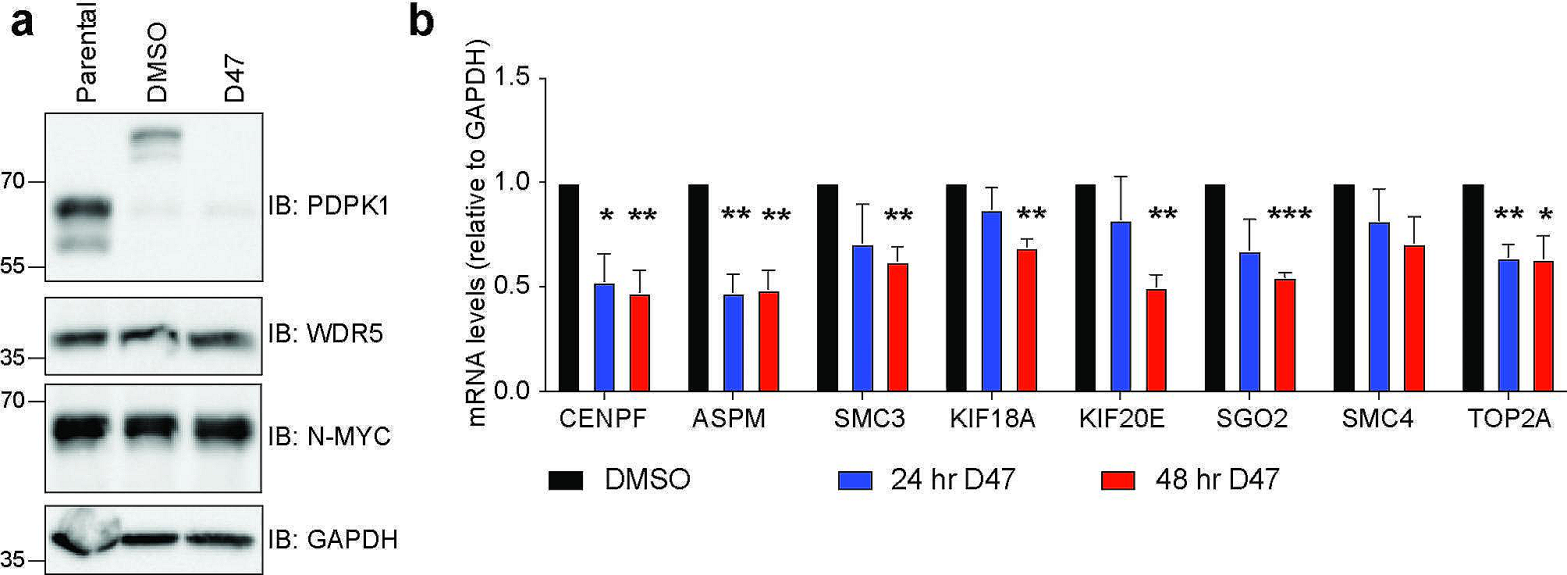
Depletion of PDPK1 using the dTAG method reduces expression of mitotic genes. (**a**) Western blot showing PDPK1 expression in parental CHP-134 cells and cells engineered so that endogenous PDPK1 is expressed in frame with a FKBP12(F36V)-2xHA tag, treated with either DMSO or 500 nM dTAG47 (D47) for 24 h to induce PDPK1 degradation. Uncropped blots are presented in Additional File 3: Figure S1. (**b**) mRNA analysis of genes involved in mitosis following treatment of engineered CHP-134 cells for 24–48 h with 500 nM dTAG47. *GAPDH* is used for normalization as a reference gene (*n* = 3 biological replicates, error bars are standard error, ***P < 0.001, **P < 0.01, *P = 0.05 using unpaired *t*-test, two-tailed)

To understand the influence of PDPK1 on the transcriptome in N-MYC amplified neuroblastoma cells, we engineered CHP-134 cells so that endogenous PDPK1 is expressed in-frame with a FKBP12(F36V)-2xHA tag, allowing PDPK1 to be degraded by the dTAG method [17]. Using this approach, addition of 500 nM dTAG47 causes PDPK1 degradation in engineered CHP-134 cells within 24 h with no obvious impact on N-MYC or WDR5 protein levels when compared to a dimethyl sulfoxide (DMSO) control (Fig. 2a, Additional File 3: Figure S1). Next, we performed quantitative mRNA analysis following depletion of PDPK1 for 24 and 48 h to probe the expression of genes we previously identified as connected to the WDR5-PDPK1 interaction, including the kinetochore genes, *CENPE* and *CENPF*, the spindle formation regulator gene, *ASPM*, and genes involved in cohesion and chromosomal segregation which include *SMC3*, *SMC4*, and *TOP2A* [12]. At both timepoints, expression of these genes is decreased relative to vehicle-treated cells (Fig. 2b), indicating that in the N-MYC amplified CHP-134 cell line PDPK1 facilitates expression of genes connected to the spindle and kinetochore.

**Fig. 3:**
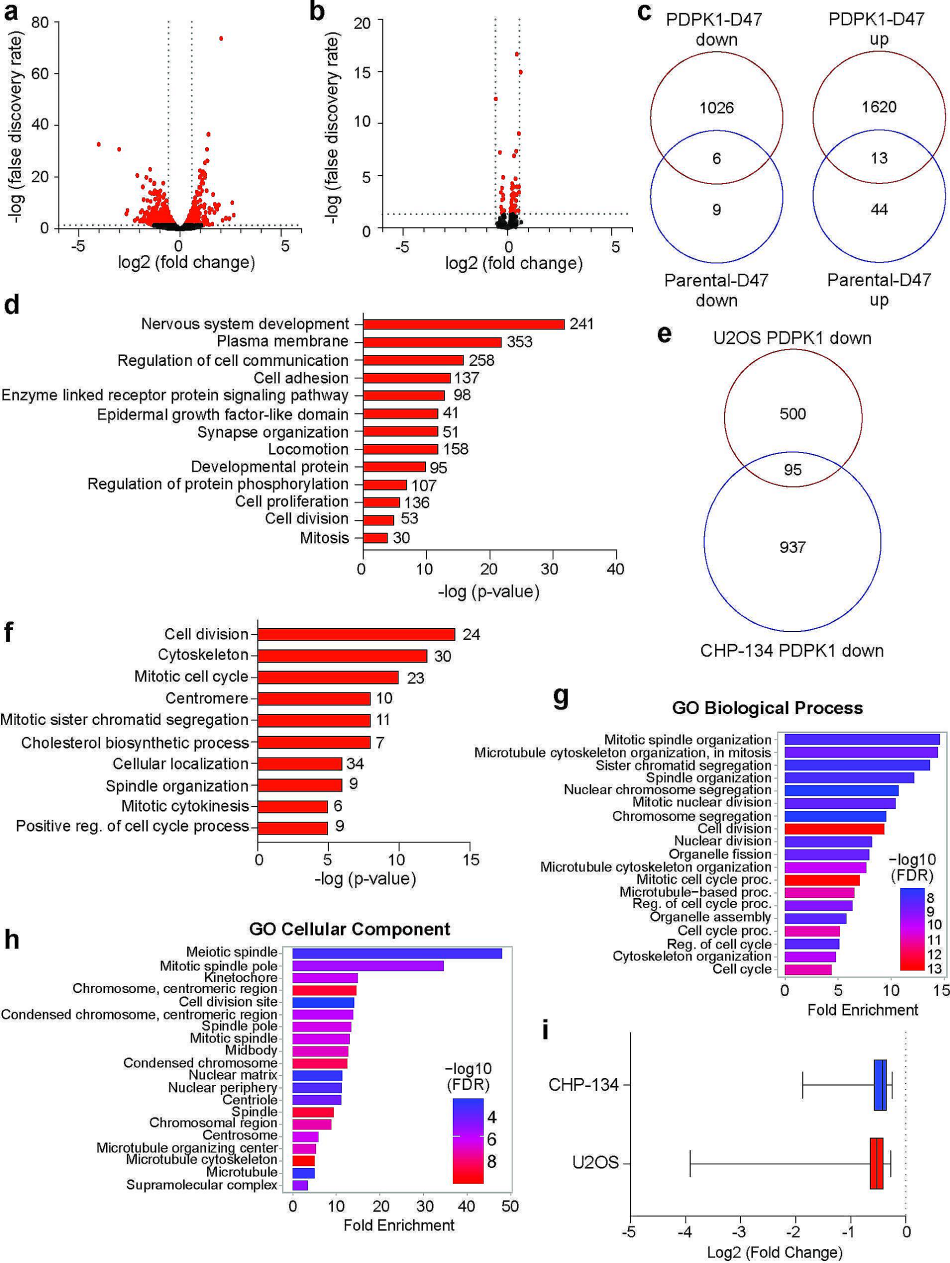
Depletion of PDPK1 using the dTAG method reduces expression of mitotic genes (**a**) Volcano plot showing data for all genes detected following depletion of PDPK1 in CHP-134 cells using 500 nM dTAG47 for 24 h. Red dots are genes that significantly changed (FDR < 0.05) and dotted lines indicate the following: FDR = 0.05 and fold change = 1.5. (**b**) Volcano plot showing data for all genes detected following treatment of parental CHP-134 cells with 500 nM dTAG47. Dotted lines are indicated as in (**a**). (**c**) Venn diagram comparing the number of genes that overlap between RNA-seq data obtained from degrading PDPK1 in CHP-134 using dTAG47 (D47) versus addition of D47 to parental CHP-134 cells. “Up” indicates genes that were significantly increased in expression while “Down” indicates genes that were significantly decreased in expression (FDR < 0.05). (**d**) Gene ontology term analysis using David Bioinformatics Resource for genes that significantly decreased in expression following degradation of PDPK1. Number of genes in each category are displayed next to the bar. (**e**) Venn diagram comparing the number of genes that significantly decrease after degrading PDPK1 for 24 h in U2OS [12] or CHP-134 cells. (**f**) Gene ontology term analysis using David Bioinformatics Resource for the 95 genes in (**e**) that are commonly decreased between U2OS and CHP-134 cells. (**g**) Gene enrichment analysis for the 95 genes in (**e**) using ShinyGO [19], GO: Biological Process. (**h**) Gene enrichment analysis for the 95 genes in (**e**) using ShinyGO [19], GO: Cellular Component. (**i**) Magnitude of change in expression for the 95 genes in (**e**) in U2OS cells and CHP-134 cells. Data are plotted as a box-and-whisker plot and line represents the median with whiskers shown at minimum and maximum points

To obtain a global view of PDPK1-dependent changes in gene expression, we performed RNA-seq on the early (24 h) timepoint samples. Approximately 2600 differentially expressed genes were detected, with roughly equal gene expression changes in either direction (Fig. 3a, Additional File 4: Table S3, Additional File 5: Figure S2a). To control for any off-target gene expression effects due to chemical addition of dTAG47, we performed RNA-seq on parental CHP-134 cells treated with 500 nM dTAG47. In the parental CHP-134 cells, 44 genes are increased in expression and 14 genes are decreased in expression, all with small fold changes (Fig. 3b, c, Additional File 5: Figure S2b) indicating that gene expression changes detected following depletion of PDPK1 are mainly specific to degradation of PDPK1. To identify the biological functions associated with PDPK1-regulated genes, we performed gene ontology (GO)-term analysis using the David Bioinformatics Resource (https://david.ncifcrf.gov/). Suppressed genes were enriched within a variety of gene functions such as neuronal development, cell adhesion, and signal transduction (Fig. 3d). In line with our targeted mRNA analysis (Fig. 2b), mitotic genes were represented among the PDPK1-suppressed genes, which was not due to any overt changes in proliferative capacity or cell cycle phase distribution (Additional File 6: Figure S3a, b), indicating that changes in mitotic gene expression are not a consequence of indirect changes to cell cycle. Additional genes that were induced following PDPK1 depletion showed enrichment among gene categories related to protein synthesis and RNA processing (Additional File 5: Figure S2c), revealing a broad spectrum of biological processes potentially regulated by PDPK1. Next, we compared PDPK1-regulated genes in CHP-134 cells to those we detected previously in the U2OS osteosarcoma cell line, a cell line that has low levels of c-MYC expression [12, 18]. In doing this analysis, we find that only 95 genes are commonly suppressed by PDPK1 across both cancer contexts (Fig. 3e). Strikingly, these genes are enriched predominantly among biological functions related to cell division and mitosis (Fig. 3f) which we separately confirmed by performing an independent gene ontology analysis using ShinyGO [19] (Fig. 3g, h). The similarities in regulation of these genes are also captured by comparison of the fold change in gene expression between CHP-134 and U2OS cells as well (Fig. 3i). Taken together, we conclude that PDPK1 predominately contributes to context-dependent gene expression, but regulation of genes related to mitosis may be a conserved function of PDPK1 regardless of cancer cell type.

### N-MYC and PDPK1 regulate common set of genes in CHP-134 cells

**Fig. 4:**
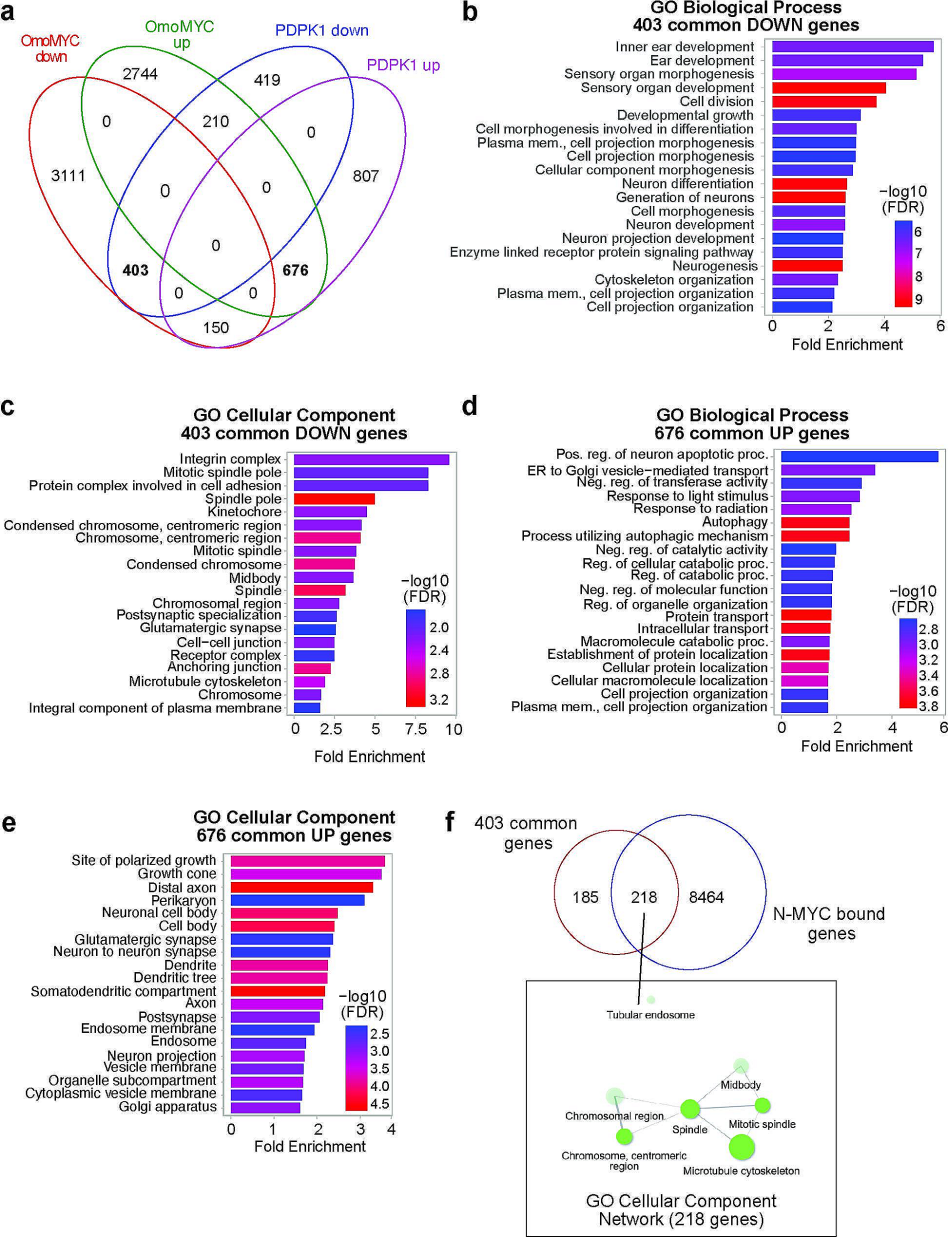
Comparison of N-MYC and PDPK1 regulated genes. (**a**) Venn diagram showing the overlap of N-MYC regulated genes as determined by OmoMYC induction in Fig. 1 and PDPK1 regulated genes as determined by depletion of PDPK1 in Fig. 3. (**b**) Gene enrichment analysis for the 403 genes in (**a**) that are commonly suppressed by inhibition of N-MYC and PDPK1 using ShinyGO, GO: Biological Process or (**c**) ShinyGO, GO: Cellular Component. (**d**) Gene enrichment analysis for the 676 genes in (**a**) that are commonly induced by N-MYC and PDPK1 using ShinyGO, GO: Biological Process or (**e**) ShinyGO, GO: Cellular Component. (**f**) Venn diagram showing the overlap of the 403 genes commonly suppressed by inhibition of N-MYC and PDPK1 compared to N-MYC bound genes detected previously in CHP-134 cells using ChIP-seq [14]. Gene ontology analysis of the 218 N-MYC targets is shown as a network below the overlap region to emphasis gene categories observed

Based on our gene expression experiments, both N-MYC and PDPK1 can separately control mitotic gene expression in N-MYC amplified cells. To identify all commonly regulated genes between N-MYC and PDPK1, we compared differentially expressed genes from the OmoMYC analysis to those obtained following depletion of PDPK1. We find that there are 403 genes that are commonly suppressed following OmoMYC induction and PDPK1 depletion, while 676 genes are commonly induced (Fig. 4a). Gene ontology analysis of the 403 commonly suppressed genes shows these genes enrich among functions related to development and differentiation (Fig. 4b, GO: Biological Process) and those related to mitosis such as spindle pole, kinetochore, and chromosome condensation and segregation (Fig. 4c, GO: Cellular Component). Of the 403 genes, the gene category showing the highest enrichment is the “integrins”, which encode an essential group of proteins involved in cell adhesion, an important cellular function that needs to be dynamically modulated during mitosis [20]. Of the 676 genes that are commonly induced, they are modestly enriched within gene categories related to organelle function and transport, polarization, and neuronal processes (Fig. 4d, e), with no obvious connections to any one particular cellular function. Because the 403 commonly suppressed genes show more prominent clustering within informative gene ontology terms, we analyzed how many of these genes are bound by N-MYC in CHP-134 cells by comparing them directly to the genes annotated to N-MYC chromatin binding sites in our previous ChIP-seq analysis [14]. Over 50% of the 403 genes show binding of N-MYC and gene ontology analysis reveals that N-MYC-bound genes are enriched mainly in gene categories related to mitosis (Fig. 4f), suggesting that a large proportion of the genes commonly regulated by N-MYC and PDPK1 are direct N-MYC targets in CHP-134 cells.

### The N-MYC-PDPK1-WDR5 nexus and regulation of mitotic gene expression

**Fig. 5:**
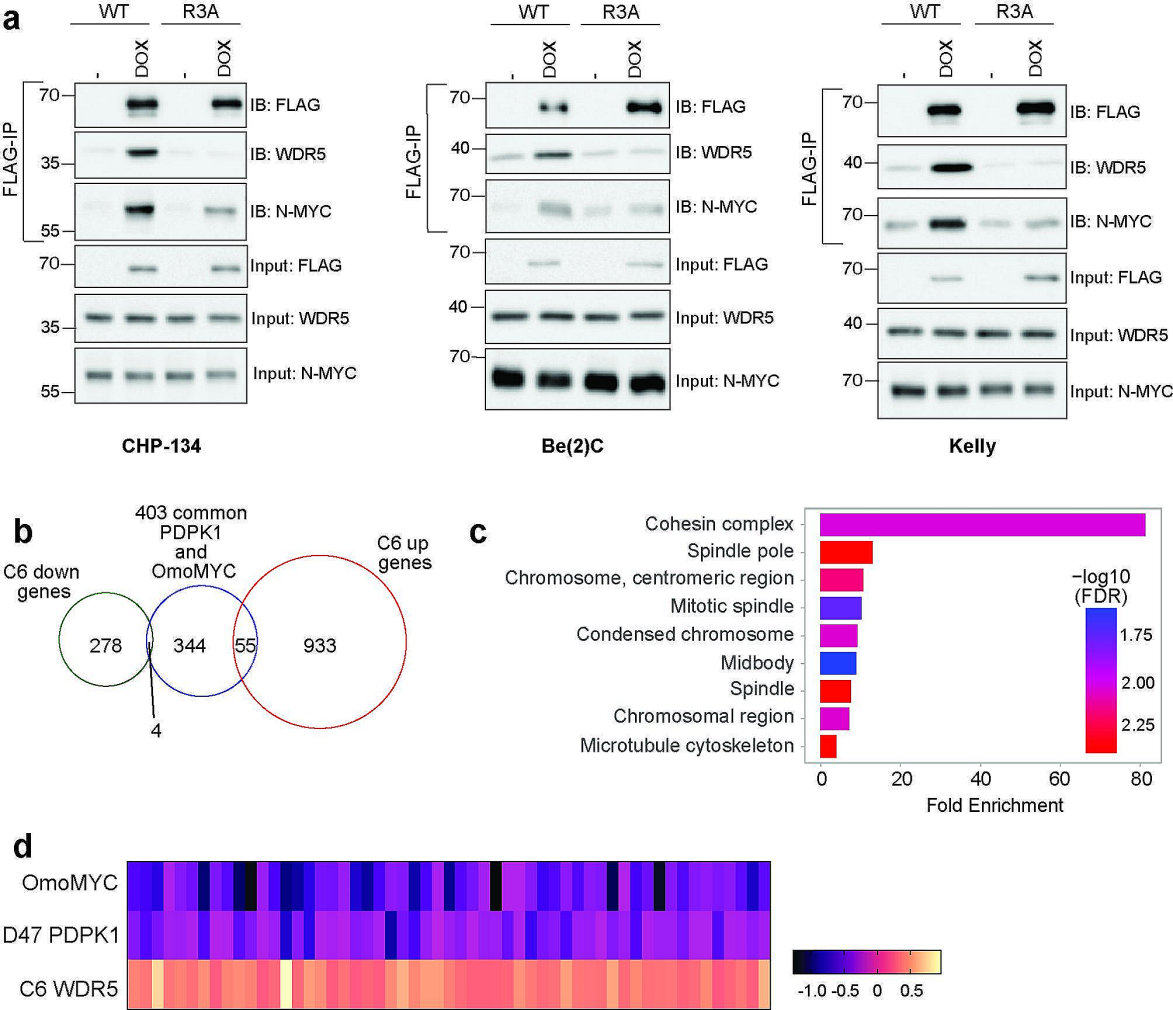
N-MYC, PDPK1, and WDR5 mediated regulation of mitotic genes. (**a**) Western blot showing proteins co-immunoprecipitated following induction of wild-type (WT) FLAG-epitope tagged PDPK1 in indicated cells compared to that of FLAG-epitope tagged PDPK1 with a point mutation that prevents direct binding of WDR5 (R3A). DOX indicates samples in which PDPK1 expression is induced using doxycycline. When WDR5 cannot interact with PDPK1, N-MYC binding is reduced. A similar effect is also observed across all N-MYC amplified cell lines (*n* = 3 biological replicates per cell line). Uncropped blots are presented in Additional File 3: Figure S1. (**b**) Comparison of 403 genes that are commonly regulated by N-MYC and PDPK1 to genes regulated in either direction following WDR5 WIN-site inhibition in CHP-134 cells using the small molecule C6 [21]. (**c**) Gene enrichment analysis for the 55 genes in (**b**) that are commonly regulated by N-MYC, PDPK1, and WDR5 using ShinyGO, GO: Cellular Component. (**d**) Heatmap showing log2(fold change) for the 55 genes in (**b**) that are commonly regulated by N-MYC, PDPK1, and WDR5

So far, our data support the idea that both the N-MYC oncoprotein transcription factor and the master kinase PDPK1 promote expression of genes linked to mitotic cell division and more specifically those involved in chromosome segregation. We next investigated the potential of this common regulation to be associated with a common protein interaction partner that N-MYC and PDPK1 both share—WDR5. WDR5 interacts with a vast number of protein interaction partners through one of two interaction surfaces. N-MYC binds directly to WDR5 through the WDR5-binding motif (WBM) site and this interaction controls target gene selection by N-MYC [14]. PDPK1 on the other hand binds directly to WDR5 through a high affinity variant of the WDR5-interaction motif (WIN) site and this interaction modulates transcription and expression of genes linked to spindle pole and kinetochore formation [12]. To determine WDR5 can serve as a scaffold for the co-binding of both N-MYC and PDPK1, we engineered CHP-134 cells to inducibly express a FLAG-epitope tagged wild-type PDPK1 (WT) or a version of PDPK1 that cannot bind WDR5 (R3A). Following induction and immunoprecipitation of WT- or R3A-PDPK1, co-immunoprecipitated material was probed for WDR5 and N-MYC. As shown in Fig. 5a (Additional File 3: Figure S1), the interaction of WDR5 with PDPK1 is inhibited by the R3A mutation, consistent with the third arginine of PDPK1 being a critical residue for the direct WDR5-PDPK1 interaction [12]. The levels of N-MYC co-immunoprecipitated with PDPK1 also are reduced by the R3A mutation, indicating that PDPK1 physically interacts with N-MYC at least in part through the WDR5-PDPK1 interaction, which again is not due to any overt changes in cell cycle phase distribution or impact of expression on cellular proliferation (Additional File 6: Figure S3c, d). To investigate whether the molecular interaction between N-MYC, PDPK1, and WDR5 is observed in other N-MYC amplified neuroblastoma cell lines we also engineered Kelly and Be(2)C cells and performed similar immunoprecipitation experiments following induction of WT- or R3A-PDPK1. In both these cell lines, the interaction of WDR5 with PDPK1 is inhibited by the R3A mutation with a concomitant decrease in N-MYC binding (Fig. 5a, Additional File 3: Figure S1), indicating that across multiple N-MYC amplified neuroblastoma cell lines N-MYC can associate with PDPK1 through their shared direct interactions with WDR5.

To understand how WDR5 impacts the expression of N-MYC and PDPK1 commonly regulated genes we specifically focused on the 403 genes that are commonly suppressed in CHP-134 cells by either genetic inhibition of N-MYC (Fig. 1) or depletion of PDPK1 (Fig. 3). These genes were compared to gene expression data previously analyzed in CHP-134 cells following 24 h treatment of cells with the chemical WIN-site inhibitor, C6 [21], which inhibits the interaction of PDPK1 with WDR5 [12]. Comparison of these three gene expression data sets reveals that of the 403 genes commonly suppressed by the inhibition of PDPK1 and N-MYC, only 59 of these genes are also differentially expressed following C6 treatment and most of those (55 genes) increase in expression (Fig. 5b). Interestingly, even though the total number of genes are small, the 55 genes are heavily enriched within gene categories related to mitotic processes, with large fold enrichments for some categories such as the cohesins (Fig. 5c). However, direct analysis of the magnitude of change individually across all 55 genes illustrates the differences in gene regulation by N-MYC, PDPK1 and WDR5 (Fig. 5d) and suggests that WDR5, in contrast to N-MYC and PDPK1, has an antagonist role in the expression of these types of genes.

## Discussion

In this study, we focus on the intersection of N-MYC with two cellular regulators that have known ties to cell cycle processes—PDPK1 and WDR5—to understand how each contributes to mitotic gene regulation in neuroblastoma cells marked by overexpression of N-MYC. We find that consistent with known functions of MYC family members [22], N-MYC controls expression of diverse sets of genes and acts as an activator of mitotic gene regulation (Fig. 1). Analysis of how PDPK1 influences the N-MYC amplified cell transcriptome reveals much less diversity in types of genes controlled by PDPK1 with those involved in cell development, signaling, and mitotic processes being the most obvious gene categories (Fig. 3). Interestingly, comparison of PDPK1-regulated genes to previous data collected in U2OS cancer cells shows that genes related to spindle pole function and chromosome segregation are conserved genes regulated by PDPK1 regardless of cancer context (Fig. 3). U2OS cells are known to express low levels of endogenous c-MYC [18] and therefore the overlap in these conserved genes suggest that the ability of PDPK1 to regulate mitotic gene expression is not an acquired function driven by over expression of N-MYC.

PDPK1 is a master kinase most well-known for its role in functioning within the phosphoinositide 3-kinase (PI3K)-AKT pathway [23], however PDPK1 can also promote oncogenesis through its ability to phosphorylate and signal to Polo-like kinase 1 (PLK1) which in turn can increase MYC stability [24, 25] and cell cycle progression [26]. Based on our data, depletion of PDPK1 in CHP-134 cells does not impact steady-state protein levels of N-MYC (Fig. 2a) as has been seen for shRNA-mediated knockdown or chemical inhibition of PLK1 in neuroblastoma cells [25]. As such we do not think that removal of PDPK1 in CHP-134 cells is impacting mitotic gene expression through its role within the PDPK1-PLK1-MYC pathway. Therefore, what we find here may be a unique arm of regulation through which PDPK1 can exert its influence on MYC activity thereby coupling signal transduction to expression of genes needed for cell growth and division. There are still many unknown questions that will need to be answered to fully understand the mechanism by which the molecular interaction between N-MYC, PDPK1, and WDR5 influences gene regulation. One outstanding question involves determining if and how growth factor signaling impacts the N-MYC-PDPK1-WDR5 connection. Based on our previous studies, inhibition of PDPK1 kinase activity does not impact the PDPK1-WDR5 interaction and small molecule WIN site inhibition does not impact phosphorylation of known PDPK1 substrates [12], suggesting that the interactions of PDPK1 with nuclear WDR5 and N-MYC may be separable from any canonical signaling pathway. Future studies focused on linking the observations from this study to growth factor signaling should be very informative.

Based on the multiple genomic comparisons in the present study, what we can conclude is that N-MYC, PDPK1, and WDR5 each control expression of genes linked specifically to spindle pole formation and chromosome segregation. However, while N-MYC and PDPK1 each act as activators for expression of these genes, inhibition of WDR5 leads to an induction of expression (Fig. 5b, c), an effect that was also observed as an early and direct transcriptional response to depletion of WDR5 [12]. Taken together with the evidence that N-MYC can interact physically with PDPK1 through the WDR5-PDPK1 interaction (Fig. 5a), we posit that N-MYC and PDPK1 may normally function to promote expression of genes involved in mitotic processes, but that if WDR5 is incorporated as an interaction partner, WDR5 acts to temper this activity. If true, this would imply that WDR5 is the key protein that ultimately dictates the level of gene expression for these groups of mitosis-related genes. It is likely that WDR5-containing protein interactions and/or complexes are in themselves cell cycle specific, as has been shown for the WDR5-KIF2A interaction and more recently the WDR5-EMBOW interaction [27], and is consistent with the role of WDR5 in mitotic bookmarking [28]. Our study was performed in asynchronous CHP-134 cells but more detailed studies to determine the types of WDR5 interactions that occur at each phase of the cell cycle, along with the types of genes regulated at each phase, could provide some insight into how and when WDR5 selects for its interaction partners during cell cycle progression.

## Conclusions

Overall, our study set out to investigate the N-MYC-WDR5-PDPK1 nexus and how it influences mitotic gene regulation in cells with MYC overexpression. Using a variety of genetically engineered N-MYC amplified neuroblastoma cell lines, we identified a small group of genes highly enriched within functional gene categories related to mitotic processes that are commonly regulated by N-MYC, WDR5, and PDPK1. We also provide evidence that a tripartite interaction between these three proteins is present in N-MYC amplified cells and that N-MYC achieves this binding, at least in part, through the WDR5-PDPK1 interaction. This study highlights the intricate and complex nature of mitotic gene regulation in cancer cells marked by MYC amplification and provides a foundation for future studies to determine the exact mechanism by which N-MYC, WDR5, and PDPK1 converge on cell cycle related processes.

## Methods

### Cell culture and lentiviral transductions

CHP-134 and Kelly cells were obtained from Sigma and HEK293T cells are in-house stocks. Be(2)C cells were a gift from Dr. Dai Chung. All neuroblastoma cells were maintained in RPMI with l-glut (Corning) containing 1% Penicillin/Streptomycin and 10% fetal bovine serum (FBS). HEK293T cells were maintained in DMEM (Corning) containing 1% Penicillin/Streptomycin and FBS. Cell lines and engineered derivatives are analyzed regularly for cancer cell integrity using STR profiling (ATCC) and tested free for mycoplasma contamination (Mycoplasma PCR Detection Kit, MP Biomedicals). In order to generate lentiviral particles for engineering neuroblastoma Tet-inducible cell lines, HEK293T cells were transfected with either EGFP, OmoMYC, wild-type PDPK1 or R3A-PDPK1 transfer plasmids (see “plasmid construction”), along with the psPAX2 packaging plasmid and the pMD2.G envelope plasmid as described previously [16]. Supernatant containing virus particles was collected in normal neuroblastoma cell maintenance media and used to transduce the CHP-134, Kelly, and Be(2)C cell lines. Transductions were performed over two days with an additional recovery day prior to selection. Selection in 0.6 mg/ml G418 (Sigma) was performed over three to seven days and then normal maintenance media was replaced with media containing Tet-approved FBS (Takara Bio) instead of normal FBS for the remainder of the experiments.

### Creation of TET-inducible expression constructs

Tet-inducible lentiviral transfer vectors containing EGFP and OMOMYC were created previously [16]. Those containing wild-type PDPK1 or R3A-PDPK1 were generated through PCR amplification of PDPK1 or R3A PDPK1 from an expression plasmid [12] and insertion into the multiple cloning site of pENTR1A donor vector with a Flag-epitope tag via Gibson assembly. Each was then inserted into the lentiviral pInducer20 acceptor vector using Gateway cloning. pENTR1A no ccDB (w48-1) was a gift from Eric Campeau & Paul Kaufman (Addgene plasmid # 17,398) [29] and pInducer20 was a gift from Stephen Elledge (Addgene plasmid # 44,012) [30].

### Endogenous PDPK1 cell line engineering

The FKBP12(F36V)-P2A-BFP or FKBP12(F36V)-P2A-mCherry targeting vectors used to insert the FKBP12(F36V)-2xHA cassette into the PDPK1 locus were made previously [16]. These vectors were delivered to CHP-134 cells along with the Cas9-sgRNA ribonucleoprotein complexes to target PDPK1 for C-terminal tagging using the Neon Electroporation Transfection System (Invitrogen) as previously described [12]. Each electroporation reaction contained 30 pmol sgRNA and 10 pmol Cas9 with 600,000 CHP134 cells, 12.5 µg FKBP12(F36V)-P2A-BFP or FKBP12(F36V)-P2A-mCherry targeting vectors vectors (1:1 mCherry: BFP) and 35 µl Neon Buffer R. Conditions used for CHP134 cells were 20 ms pulse width, 1200 V, and three pulses and following this, cells were plated into warm, antibiotic-free maintenance media for two days. After a minimum of five days, viable cells double-positive for BFP and mCherry were sorted together using a BD FACSAria III instrument.

### Protein lysates and immunoprecipitation experiments

Approximately 2.0 × 10^6^ CHP-134 cells expressing a degradable version of PDPK1 were treated with 500 nM dTAG47 or matched dimethyl sulfoxide (DMSO) control for 24 h prior to cell harvesting. Protein lysates of engineered and parental CHP-134 cells were generated through lysis in ice-cold buffer containing 150 mM Tris-HCl pH 8.0, 5 mM EDTA, 150 mM NaCl, and 1% Triton X-100, 0.01 M PMSF, and protease inhibitor tablet (Roche) and brief sonication on 25% power for 15 s. Samples were centrifuged for 10 min at 13,000 RPM to clarify lysates and concentrations of lysates for each sample determined using the BioRad Bradford assay. All samples were normalized to each other and boiled for 5 min in SDS-loading dye containing beta-Mercaptoethanol prior to Western blot. For Flag-immunoprecipitations (IP) in CHP-134 cells, PDPK1 expression was induced approximately 20.0 × 10^6^ cells with 500 ng/ml doxycycline for 24 h. For Kelly and Be(2)C cell lines, PDPK1 expression was induced in approximately 7.5 × 10^6^ cells using 0.5–1 µg/ml doxycycline for 24 h. Doxycycline was sterile-filtered and made weekly to ensure integrity. Following cell lysis as described above, lysates were normalized, and a small fraction of the lysate was kept as “input”. Remaining protein lysate was used for Flag-IP by adding M2(Flag)-conjugated agarose beads (Sigma) overnight at 4 °C. The next day, M2(Flag)-beads were blocked using 1% bovine serum albumin in normal lysis buffer for 30 min and then added to all IP samples and allowed to rotate for 3 h at 4 °C. M2(Flag)-beads were washed four times in cold lysis buffer for a total of 5 min each wash prior to transfer to a new tube and boiling in 2.5X SDS-loading dye containing beta-Mercaptoethanol. Input samples were diluted to match the IP sample volume and proteins analyzed using Western blot analysis.

### Western blotting and antibodies

All proteins were resolved by SDS-PAGE and transferred to a PVDF membrane (PerkinElmer). Membranes were blocked in TBS-T (50 mM Tris, pH 7.5, 0.1% Tween-20, 150 mM NaCl) containing 5% milk. Following blocking, primary antibodies were added in block solution overnight at 4 °C on a rocker. Multiple antibodies were used for some original blots shown in Additional File 3: Figure S1 if there could be at least two ladder markers still be present around each protein of interest. For blots that were trimmed, images from all replicates are included. The next day, all blots were washed three times in TBS-T for 5 min each wash and secondary antibodies were added for 1 h at room temperature. Following an additional three washes for 5 min each, images were obtained on a Bio-Rad ChemiDoc MP instrument using the Clarity ECL substrate. Antibodies used for immunoblotting were as follows: PDK1 (used for PDPK1, Cell Signaling, 13,037), N-MYC (Cell Signaling, 51,705), WDR5 (Cell Signaling, 13,105), Flag-HRP (Cell Signaling, 86,861), GAPDH-HRP (Cell Signaling, D16H11).

### Cell proliferation and cell cycle analysis

2.0 × 10^6^ engineered PDPK1 CHP-134 cells or parental CHP-134 cells were treated with DMSO or 500 nM dTAG47 for 24 h. The WT versus R3A analysis was performed similarly, except cells were induced with 500 ng/ml freshly made doxycycline at time of plating. At 24 h post-treatment, total cell counts were determined for all samples and 1.0 × 10^6^ cells per sample was pelleted and resuspended in ice-cold 70% ethanol before storage at -20 °C. To prepare cells for cell cycle analysis, each sample was thawed and centrifuged at 800 x g for 6 min. Cell pellets were washed gently in 1X phosphate buffered saline (PBS) and again centrifuged at 800 x g for 6 min. Pellets were resuspended in PBS containing 10 µg/ml propidium iodide, 100 µg/ml RNAse A, and 2 mM MgCl_2_ and allowed to stain overnight at 4 °C. All stained cells were filtered through a 35 μm nylon mesh cell strainer prior to being analyzed on a Guava easyCyte Flow Cytometer instrument (Luminex). A minimum of 10,000 cells were recorded for each sample and single cells were selected for analysis using forward and side scatter.

### RNA-seq and mRNA analysis

To deplete PDPK1, 2.0 × 10^6^ engineered CHP-134 cells were treated with 500 nM dTAG47 or matched DMSO for 24–48 h prior to cell harvesting. At this time, Trizol (Invitrogen) was added to collect cells and RNA was extracted with the Direct-zol RNA mini-prep kit (Zymo Research) following manufacturer’s instructions. For RT-qPCR mRNA analysis, cDNA was generated from purified RNA using MulV reverse transcriptase (Promega) and random hexamers. cDNA that resulted was analyzed using a AriaMx Real-Time PCR Machine (Agilent) with *GAPDH* used as a reference (normalization) gene. Primers for genes used in this study were listed previously [12]. For RNA-seq, 2 µg of purified RNA following 24 h timepoint was submitted to Vanderbilt Technologies for Advanced Genomics (VANTAGE) core at Vanderbilt University Medical Center. Following ribosomal RNA depletion and library generation, samples were sequenced on an Illumina NovaSeq6000 instrument. Two biological replicates were analyzed for depletion of PDPK1. Parental CHP-134 cells treated for 18 h with either 500 nM dTAG47 or DMSO control were similarly processed and submitted to VANTAGE for library generation and sequencing. Four biological replicates were analyzed for this experiment. For RNA-seq to look at N-MYC-regulated genes, 1.0 × 10^6^ CHP-134 cells engineered to express inducible EGFP and OmoMYC were treated with 1 µg/ml doxycycline and after three days RNA extracted and purified from the cells as described above. 2 µg of purified RNA was submitted to VANTAGE and samples were sequenced on an Illumina NovaSeq6000 instrument. Three biological replicates were analyzed for this experiment.

#### RNA-seq analysis

Raw sequencing data were subjected to quality control checks using FastQC (v0.11.8). For PDPK1 depletion RNA-seq analysis, adapter trimming was done using Trim Galore (0.6.4_dev, http://www.bioinformatics.babraham.ac.uk/projects/trim_galore/) which also removed any low quality reads. Reads were then aligned to the hg19 human genome using STAR alignment tools [31] followed by quantification of gene expression using FeatureCounts [32]. Gene count matrices were generated from the aligned reads, which represent the expression levels of genes in each sample. Differentially expressed genes were identified using R package DESeq2 [33]. Significantly differentially expressed genes were identified based on adjusted p-values (FDR) and fold change. Benjamini-Hochberg procedure applied to control false discovery rates from multiple testing. Significantly changed genes were called with a FDR < 0.05. For remaining RNA-seq analysis, similar approach was used for hg19 genome alignment and quantification and adapter trimming was done using Cutadapt [34]. Differential analysis was performed also using DESeq2 [33], and adjusted p-values (FDRs) were determined by the Benjamini-Hochberg procedure. Significantly changed genes were called with a FDR < 0.05.

### Electronic supplementary material

Below is the link to the electronic supplementary material.

Supplementary Material 1

Supplementary Material 2

Supplementary Material 3

Supplementary Material 4

Supplementary Material 5

Supplementary Material 6

Supplementary Material 7

## Data Availability

Sequencing data in this study are deposited on GEO under GSE244835 and GSE244899. RNA-seq data for depletion of PDPK1 in U2OS cells was previously published under GSE150400 [12]. N-MYC ChIP-seq data was previously published under GSE222157 [14] and RNA-seq data for C6 WIN-site WDR5 treatment was previously published under GSE136451 [21]. Additional data are available upon request.
