# Supplementary material for: Mitotic gene regulation by the N-MYC-WDR5-PDPK1 nexus
Source: BMC Genomics. 2024 Apr 11;25:360. doi: 10.1186/s12864-024-10282-6 (PMC11007937; doi:10.1186/s12864-024-10282-6)
Supplement: Supplementary file 1 — Supplementary Material 1 [file 12864_2024_10282_MOESM1_ESM.pdf]

Supplementary Information

Additional File 1: Table S1. RNA-seq analysis of CHP-134 cells following induction of OmoMYC compared to induction of EGFP (FDR < 0.05). Excel file showing differentially expressed genes in engineered CHP-134 cells following genetic inhibition of N-MYC.

Additional File 2: Table S2. Gene set enrichment analysis of RNA-seq data obtained following induction of OmoMYC compared to induction of EGFP (FDR < 0.05). Excel file showing GSEA results for genes differentially expressed in engineered CHP-134 cells following genetic inhibition of N-MYC.

Additional File 3: Figure S1. Uncropped Western blots that correspond to Figure 2a and Figure 5a. Pictures of uncropped Western blots. Merged images with ladder are added for reference of when cropping occurred.

Additional File 4: Table S3. RNA-seq analysis of engineered CHP-134 cells treated with 500 nM dTAG47 for 24 hr to induce degradation of PDPK1 (FDR < 0.05). Excel file showing differentially expressed genes in engineered CHP-134 cells following acute depletion of PDPK1.

Additional File 5: Figure S2. Additional RNA-seq analysis for depletion of PDPK1 in CHP-134 cells. (a) Heatmap showing normalized read count data for significantly changed genes (FDR < 0.05) obtained from degrading PDPK1 in CHP-134 using 500 nM dTAG47 for 24 hr, compared to DMSO control. (b) Heatmap showing normalized read count data obtained from treating parental CHP-134 cells with 500 nM dTAG47 for 18 hr, compared to DMSO control. (c) Gene ontology term analysis using David Bioinformatics Resource for genes that significantly

increased in expression following degradation of PDPK1. Number of genes in each category are displayed next to the bar.

Additional File 6: Figure S3. Impact of genetic perturbations on cell proliferation and cell cycle phase distribution. (a) Equal numbers of parental or engineered PDPK1 CHP-134 (DT-PDPK1) cells were plated with DMSO or 500 nM dTAG47 (D47) for 24 hr and then total cell counts determined. Bar graph shows mean calculated for each sample (*n* = 3 biological replicates, error bars are standard error). (b) Cells in (a) were collected for cell cycle phase distribution analysis and results are shown for each cell line with or without dTAG47 treatment for 24 hr (*n* = 3 biological replicates, error bars are standard error). (c) Equal numbers of engineered CHP-134 cells were induced with 500 ng/ml doxycycline to express either Flag-epitope tagged wild-type PDPK1 (WT) or a version of PDPK1 that cannot bind WDR5 (R3A). After 24 hr, total cell counts were determined and bar graph shows mean calculated for each sample (*n* = 3 biological replicates, error bars are standard error). (d) Cells in (c) were collected for cell cycle phase distribution analysis and results are shown for each cell line (*n* = 3 biological replicates, error bars are standard error).
