# Supplementary material for: Mitotic gene regulation by the N-MYC-WDR5-PDPK1 nexus
Source: BMC Genomics. 2024 Apr 11;25:360. doi: 10.1186/s12864-024-10282-6 (PMC11007937; doi:10.1186/s12864-024-10282-6)

Uncropped blot Figure 2a

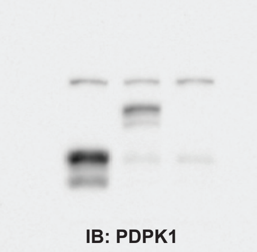

Uncropped blot Figure 2a

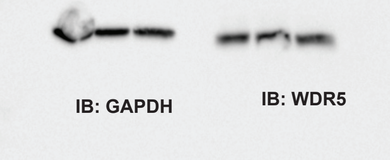

Uncropped blot Figure 2a

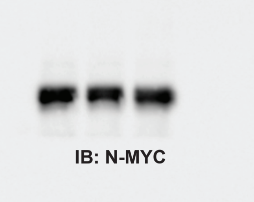

Uncropped blot Figure 2a
with Ladder merge (cropped at ladder marks)

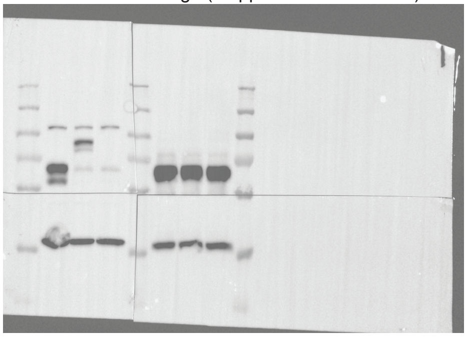

Uncropped blot Figure 5a

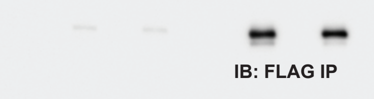

Uncropped blot Figure 5a

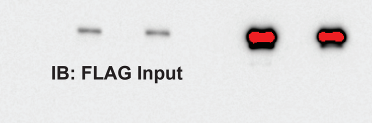

Uncropped blot Figure 5a, CHP-134

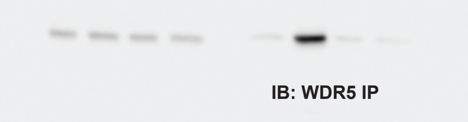

Uncropped blot Figure 5a, CHP-134

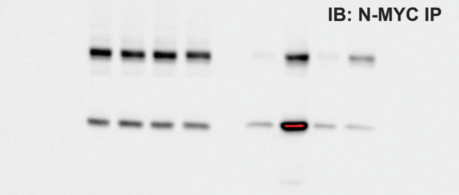

Uncropped blot Figure 5a, CHP-134

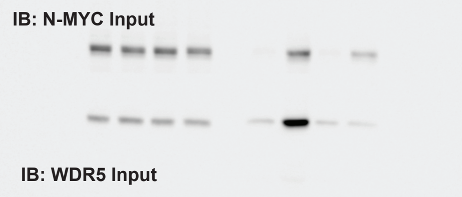

Uncropped blot Figure 5a (CHP-134)
with Ladder merge for N-MYC and WDR5 antibody
(cropped at ladder marks) replicate 1

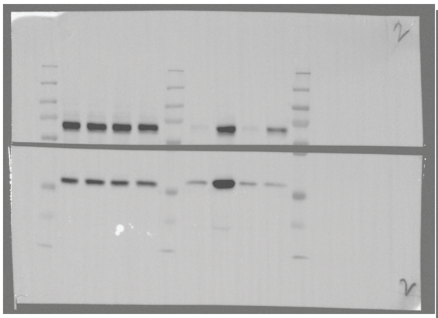

Uncropped blot Figure 5a (CHP-134)
with Ladder merge for FLAG antibody
(cropped at ladder marks) replicate 1

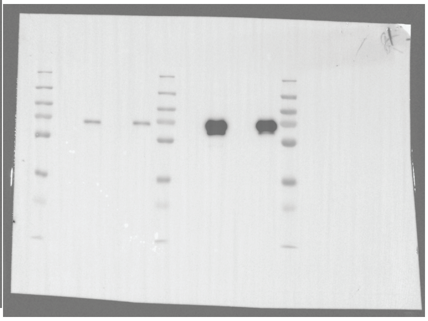

Uncropped blot Figure 5a (CHP-134)
with Ladder merge for FLAG antibody
(cropped at ladder marks) replicate 3

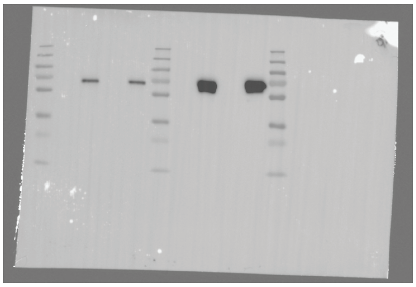

Uncropped blot Figure 5a (CHP-134)
with Ladder merge for FLAG antibody
(cropped at ladder marks) replicate 2

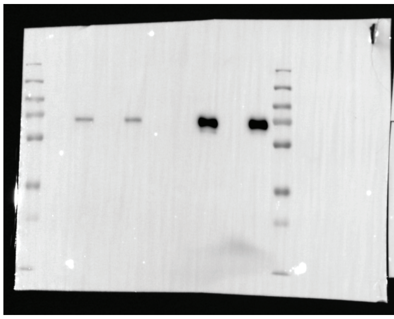

Uncropped blot Figure 5a (CHP-134)
with Ladder merge for N-MYC and WDR5 antibody
(cropped at ladder marks) replicate 2

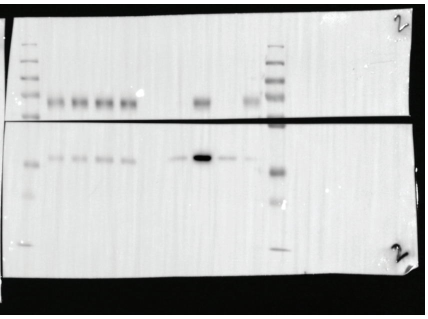

Uncropped blot Figure 5a (CHP-134)
with Ladder merge for N-MYC and WDR5 antibody
(cropped at ladder marks) replicate 3

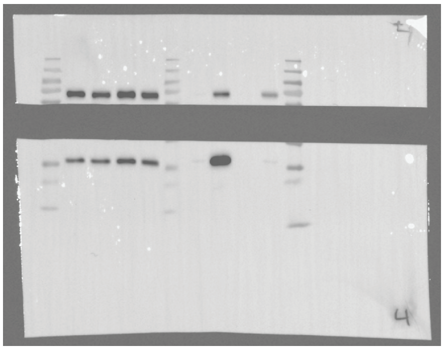

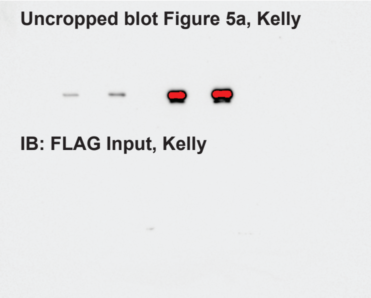

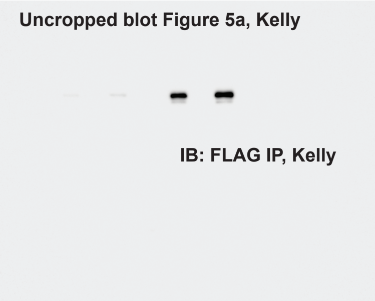

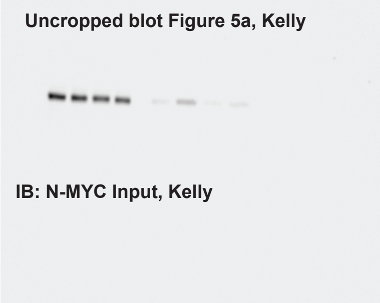

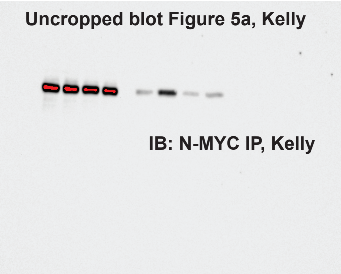

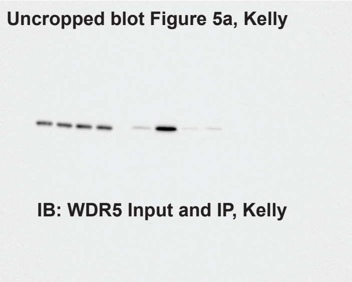

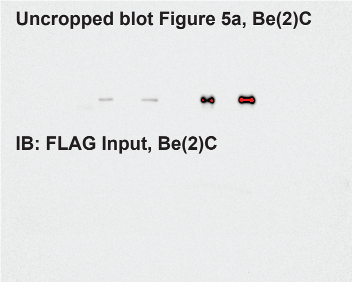

Uncropped blot Figure 5a (Kelly)
with Ladder merge for FLAG antibody

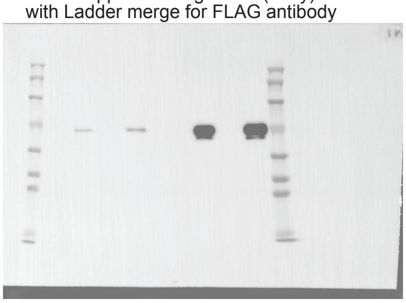

Uncropped blot Figure 5a Kelly
with Ladder merge for N-MYC antibody

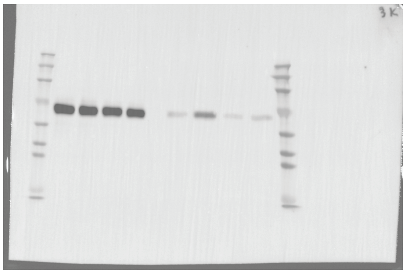

Uncropped blot Figure 5a Kelly
with Ladder merge for WDR5 antibody

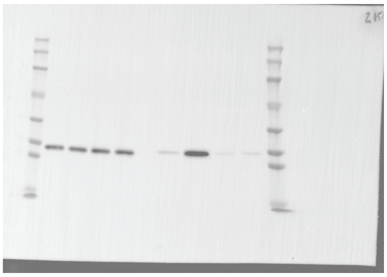

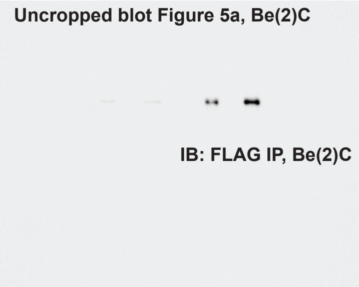

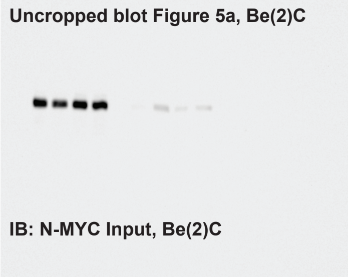

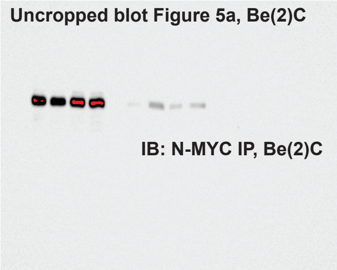

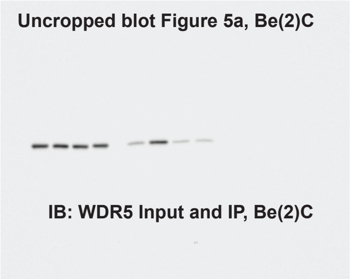

Uncropped blot Figure 5a (Be(2)C)
with Ladder merge for FLAG antibody

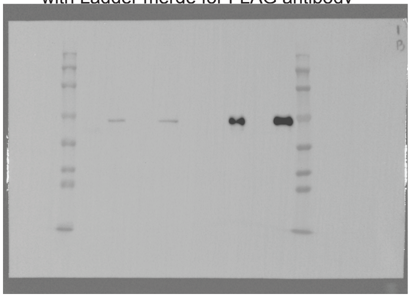

Uncropped blot Figure 5a (Be(2)C)
with Ladder merge for N-MYC antibody

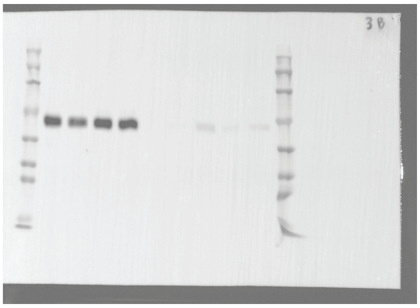

Uncropped blot Figure 5a (Be(2)C)
with Ladder merge for WDR5 antibody

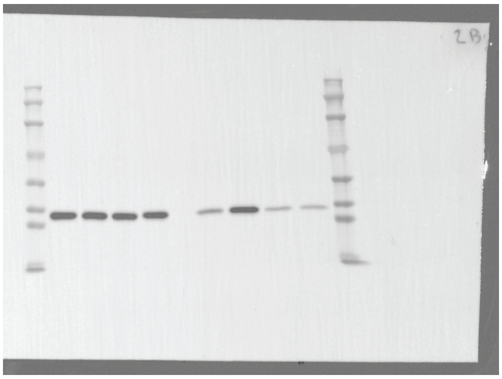

Supplement: Supplementary file 4 — Supplementary Material 4 [file 12864_2024_10282_MOESM4_ESM.pdf]
