# Supplementary figures and images for: Mitotic gene regulation by the N-MYC-WDR5-PDPK1 nexus
Source: BMC Genomics. 2024 Apr 11;25:360. doi: 10.1186/s12864-024-10282-6 (PMC11007937; doi:10.1186/s12864-024-10282-6)

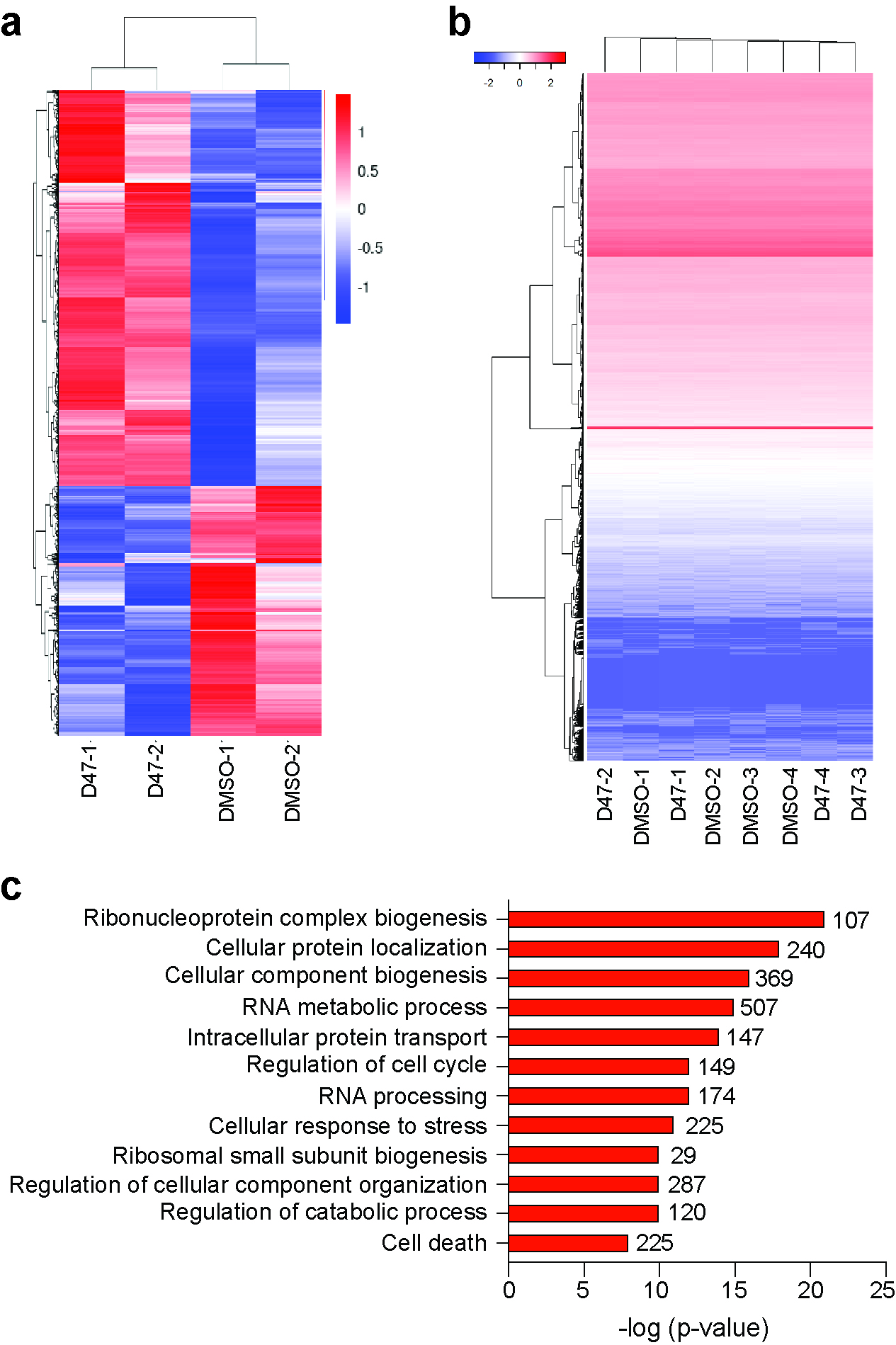

Supplement: Supplementary file 6 — Supplementary Material 6 [file 12864_2024_10282_MOESM6_ESM.jpg]

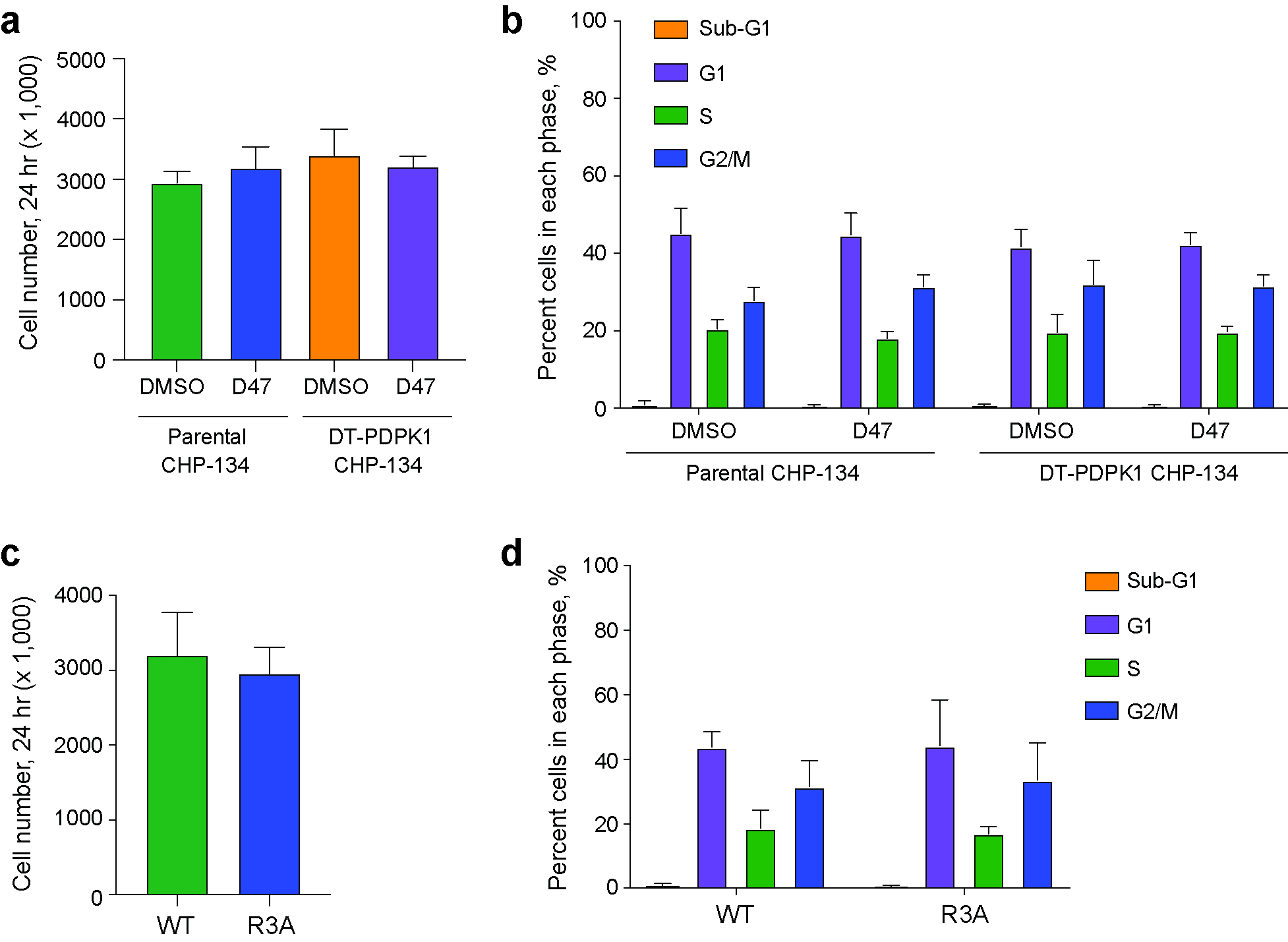

Supplement: Supplementary file 7 — Supplementary Material 7 [file 12864_2024_10282_MOESM7_ESM.jpg]
